# Adult pertussis is unrecognized public health problem in Thailand

**DOI:** 10.1186/s12879-016-1357-x

**Published:** 2016-01-25

**Authors:** Nirada Siriyakorn, Pornvimol Leethong, Terapong Tantawichien, Saowalak Sripakdee, Anusak Kerdsin, Surang Dejsirilert, Leilani Paitoonpong

**Affiliations:** 1 Division of Infectious Diseases, Department of Medicine, Faculty of Medicine, Chulalongkorn University, Rama 4 Road, Bangkok, 10330 Thailand; 2 Department of Medicine, Maharat Nakhon Ratchasima Hospital, Nakhon Ratchasima, Thailand; 3 Department of Medicine, Samutprakarn Hospital, Samutprakarn, Thailand; 4 National Institute of Health, Ministry of Public Health, Bangkok, Thailand; 5 Faculty of Public Health, Kasetsart University Chalermphrakiat Sakon Nakhon Province Campus, Sakon Nakhon, Thailand; 6 Division of Infectious Diseases, Department of Medicine, King Chulalongkorn Memorial Hospital, Bangkok, Thailand

**Keywords:** Pertussis, Chronic cough

## Abstract

**Background:**

Although pertussis has been considered a disease of childhood, it is also recognized as an important respiratory tract infection in adolescents and adults. However, in countries with routine vaccination against pertussis with high coverage, pertussis is not usually taken into consideration for the etiology of prolonged cough in adults. Previous studies in a variety of populations in developed countries have documented that pertussis is quite common, ranging from 2.9 to 32 % of adolescents and adults with prolonged cough. The anticipation and early recognition of this change in the epidemiology is important because the affected adolescents and adults act as reservoirs of the disease and source of infection to the vulnerable population of infants, for whom the disease can be life threatening. We conducted a prospective study to determine the prevalence of pertussis in Thai adults with prolonged cough.

**Methods:**

Seventy-six adult patients with a cough lasting for more than 2 weeks (range, 14–180 days) were included in the present study. The data regarding medical history and physical examination were carefully analyzed. Nasopharyngeal swabs from all patients were obtained for the detection of deoxyribonucleic acid of *Bordetella pertussis* by the polymerase chain reaction (PCR) method. Paired serum samples were collected and tested for IgG antibody against pertussis toxin by using an ELISA method.

**Results:**

Of 76 adult patients, 14 patients (18.4 %) with the mean age of 59 (range, 28–85) years and the mean duration of cough of 34 (range, 14–120) days had laboratory evidence of acute pertussis infection. One patient was diagnosed by the PCR method, while the rest had serological diagnosis. Whooping cough is a significantly associated symptom of patients with chronic cough who had laboratory evidence of pertussis. (*p* < .05, odds ratio 3.75, 95 % confidence interval: 1.00,14.06)

**Conclusion:**

Pertussis is being increasingly recognized as a cause of prolonged, distressing cough among adults in Thailand. This result addresses the need of pertussis vaccination in Thai adults for preventing transmission to a high risk group such as newborn infants.

## Background

Pertussis is a highly communicable, vaccine-preventable respiratory disease caused by *Bordetella pertussis,* a fastidious Gram-negative coccobacillus with many virulence factors notable for their roles in adhesion of the bacteria to ciliated respiratory epithelium and disrupting the normal functions of ciliated epithelial cells [1]. In the present vaccine era, the overall incidence of pertussis has been reduced dramatically [2–4]. Despite high vaccination coverage, pertussis continues to be a major cause of morbidity and mortality in both developed and less developed countries. Although pertussis has traditionally been considered a disease of infants and childhood, it was well-documented in adults nearly a century ago [5–7]. During the past 15 years, previous reports have shown that the incidence of pertussis in adolescents and adults have been increasing in western countries [8, 9]. However, the shift towards an increase in pertussis incidence among adults that has been reported in some countries may be a true increase but this should be interpreted with caution owing to the much advanced development in diagnosis and the increased awareness of the disease. Most adult patients with acute pertussis infection present with prolonged non-specific cough, which most often go unrecognized by caring physicians [10]. The increased prevalence of pertussis in adults is probably due to the waning of the immunity against *B. pertussis*, which can be obtained from a natural infection or from childhood vaccination [11]. The immunity stimulated by whole-cell vaccine and acellular vaccine can remain in the body for about 6–8 years and 4–6 years or longer, respectively [12, 13]. In Thailand, the pertussis cases have continuously declined since 1977 after the implementation of Diphtheria-Tetanus-Pertussis (DTP) vaccine in the Expanded Program for Immunization (EPI). Although the number of pertussis cases per 100,000 persons has dropped from 7.25 cases in 1977 to 0.02 cases in 2007, there are some outbreaks of this disease in Thailand [14].

Although several previous studies in developed countries have shown that pertussis is one of the most common causes of prolonged cough illnesses in adults, ranging from 2.9 to 32 %, most physicians are often unaware of this disease [15–20]. Unrecognized infected adults may spread this organism to the nonimmune individuals, and the infection in some high-risk groups including young children can be more severe and potentially fatal. In Thailand, the diagnosis of pertussis in adults is often difficult and delayed because of a lack of classic symptoms and/ or low physicians' awareness, as well as unavailable laboratory diagnosis of pertussis such as culture isolation and polymerase chain reaction (PCR) test. Prolonged cough in many patients might be resolved without a clear diagnosis. Consequently, they have risk to spread the disease to a high risk group such as infants younger than 6 months. Although a cluster of pertussis cases were reported in Thailand, the prevalence of pertussis in adults with prolonged cough has never been documented [21, 22] Thus, this prospective study was carried out to determine the prevalence of pertussis in Thai adolescents and adults with prolonged cough.

## Methods

Adult patients with age of ≥ 15 years who had a cough for more than 2 weeks were recruited at King Chulalongkorn Memorial Hospital (KCMH), Bangkok, Thailand, from October 2010 to February 2011. All the data regarding the symptoms/signs of the present illness, history of vaccination, complications and medical treatment were recorded. Chest X-ray was performed for each patient. Patients were excluded if they were immunocompromised, had chronic airway diseases or other known chronic cough illness, had received medications that can cause a cough such as angiotensin-converting enzyme inhibitors, or abnormal chest X-ray.

The study protocol was approved by the Ethical Committee of the Faculty of Medicine, Chulalongkorn University, Bangkok, Thailand. The written Informed consent was obtained from each patient after the nature of study protocol had been fully explained. For the children under the age of 18, the parent or guardian of the child had provided informed consent on behalf of the child.

### Specimen collection for *B. pertussis*

Nasopharyngeal swab was obtained from each patient by using rayon-tipped swabs on aluminum shafts. *B. pertussis* DNA was detected in nasopharyngeal swab specimens by the real-time PCR method that amplified the specific genome of target bacteria. Specific primer of target region of *B. pertussis* is the upstream region of *por* gene. The result is interpreted by a gel electrophoresis technique, melting temperature (Tm), or threshold cycle (Ct) of PCR product. The real-time PCR of SYBR green is interpreted by comparing with positive and negative control. The sequences of these 2 primers were BP-B3 (5’-GGG AAG TTG ACG CTA TTG CA-3’) and BP-BF (5’-ATC GGG CAT GCT TAT GGG TGT TCA-3’). The amplicon was 260 bp in size. The bacteria that was used to standardize these PCR tests was *Escherichia coli* DMST 26008 (containing plasmid pBORDET to positive control for *B. pertussis*) or *B. pertussis* DMST 19589. Blood samples were obtained for determination of IgG antibody by using *Bordetella* PT IgG ELISA test (IBL kit; IBL international GMBH, Germany) on day of enrollment and 2 weeks later.

### Pertussis case definition

A patient was considered to have definite laboratory-confirmed *B. pertussis* infection if the PCR test of nasopharyngeal swab specimen was positive for *B. pertussis* [23]. The four-fold rising in acute and convalescent phase of serum samples (paired sera) or the agglutinin titer of ≥ 3 SD (single serum) of *Bordetella* PT IgG was considered as acute probable pertussis infection. All patients must not have evidence of *Chlamydophila pneumoniae* and *Mycoplasma pneumoniae* infection as negative results of polymerase chain reaction of the respiratory specimens.

### Statistical analysis

Frequency and percentage were used for analysis of the prevalence of pertussis in our patients with chronic cough. A Chi-squared test and Fisher’s exact test were used to compare the frequencies between the groups. Student *t*-test and Mann Whiney *U* test were used to compare continuous variables between the groups.

## Results

Seventy-six patients (age range 15–87 years) with prolonged cough (range 14–180 days) were enrolled. Fourteen patients (18.4 %) had laboratory evidence of pertussis. One patient was diagnosed by the PCR method, compatible with definite pertussis, while 13 patients had probable pertussis (Table 1). For the group of patients who had the laboratory evidence of pertussis, the age was 59 ± 16.45 years (range 28–85 years) and the duration of cough was 34.07 ± 31.52 days (range 14–120 days). Before enrollment into the present study, most of these patients were diagnosed with allergies and bronchitis. Interestingly, the whooping cough is the statistically significant associated symptom of patients who had laboratory evidence of pertussis (*p* < .05, odds ratio 3.75, 95%confidence interval 1.00–14.06). Concerning the age of the patients, it was evident that there was a high percentage of those who were 65 years and more (35.3 % of the first group and 29.0 % of the second respectively). None of our patients had ever received a boosted acellular pertussis vaccine (Table 1).

**Table 1:** Clinical characteristics and laboratory findings of patients with prolonged cough with or without laboratory evidence of pertussis

	Patients with laboratory evidence of pertussis (*n* = 14)	Patients without laboratory evidence of pertussis (*n* = 62)	*P* value	Odd Ratio	95 % CI
Age : mean ± SD	59 ± 16.45 years	54.16 ± 16.66 years	0.33	-	4.96,14.64
(range)	(28–85)	(15–87)			
age group 15–25 years	0	3(4.83 %)			
age group 26–45 years	3(21.4 %)	18(29.03 %)			
age group 46–65 years	6(48.6 %)	33(53.23 %)			
age group >65 years	5(35.7 %)	18(29.03 %0			
Male : female ratio	5:9	23:39	0.92	0.94	0.28,3.16
History of Tdap vaccination	0	0			
Duration of cough:					
mean ± SD	34.07 ± 31.52 days	30.03 ± 27.42 days	0.63	-	-
(range)	(14–120 days)	(14–180 days)			
Provisional diagnosis:					
- allergy - gastroesophageal reflux - bronchitis - bronchial asthma - post-infectious cough - others and unknown diagnosis	3 (21.4 %) 1 (7.1 %) 1 (7.1 %) 1 (7.1 %) 1 (7.1 %) 7 (50 %)	17 (27.4 %) 2 (3.2 %) 20 (32.3 %) 2 (3.2 %) 4 (6.5 %) 17(27.4 %)			
Associated symptoms:					
- weight loss - hoarseness - headache - sleep disturbance - whooping cough - Influenza-like symptoms - sneezing attack	3 (21.4 %) 6 (42.9 %) 3 (21.4 %) 3 (21.4 %) 5 (35.7 %) 4 (28.6 %) 4 (28.6 %)	11 (17.7 %) 32 (51.6 %) 16 (25.8 %) 15 (24.2 %) 8 (12.9 %) 22 (35.5 %) 22 (35.5 %)	0.71 0.55 1 1 0.04 * 0.76 0.76	1.26 0.70 0.78 0.86 3.73 0.73 0.73	0.30,5.30 0.22,2.26 0.19,3.17 0.21,3.48 1.00,14.06 0.20,2.59 0.20,2.59
Complications of cough:					
-no complication -urinary incontinence - rib fracture - pneumothorax - inguinal hernia - seizure - otitis media or hearing loss	11 (78.6 %) 1 (7.1 %) 0 0 0 0 2 (14.3 %)	49 (79 %) 11 (17.7 %) 0 1 (1.6 %) 0 0 1 (1.6 %)			

*p <0.05: statistical significant

## Discussion

There are a wide variety of diagnoses in patients with prolonged cough including the infectious cause. This pertussis-like syndrome can be caused by several viruses including adenovirus, influenza virus, parainfluenza viruses, and respiratory syncytial virus or bacteria including *Chlamydophila pneumoniae* or *Mycoplasma pneumoniae* [24]. The detection of pertussis in adolescents and adults with chronic cough has been found to be very important since the disease can transmit to infants who have no immunity to pertussis resulting in a higher mortality rate. The incidence of pertussis in adults with prolonged cough increased due to the declined level of immunity against *B.pertussis,* obtained from a childhood vaccination or natural infection. Despite high vaccine coverage in infants and children, there are resurgences of pertussis in adolescents and adults in many western countries [3, 15, 16]. Previous studies have shown that the prevalence of *B. pertussis* infection in adolescents and adults with prolonged cough of more than 1–3 weeks was 2.9 % to 32 % in developed countries [15–20]. This difference is partly because each study has different criteria of the cough duration which can range from 7 to 21 days and the laboratory diagnosis of pertussis in each study was different. Laboratory diagnostic tests for *B.pertussis* infection include the culture of the properly obtained nasopharyngeal specimen, the PCR test, and the test for serum antibodies by using enzyme-linked immunosorbent assays (ELISA) or Western blot. The present study showed that only 1.3 % of Thai adults with prolonged cough had laboratory-confirmed of *B. pertussis* infection. This is not surprising since the sensitivity to detect pertussis infection is low and depends on the timing of specimen collection. The PCR test for diagnosis of pertussis has very high sensitivity during the first 3 weeks of cough when bacterial DNA is still present in the nasopharynx [25–27]. In the present study, all patients had had cough of more than 2 weeks, and the mean duration of cough in pertussis patients was 30 days (range 14–120), and this might be too long to detect by using the PCR test.

When a variety of tests including culture, PCR and serology tests are combined in the diagnosis of pertussis in adults, the results may yield a higher prevalence of the disease. Culture of *B.pertussis* from nasopharyngeal swab has shown a high specificity but the sensitivity in the detection of this organism is relatively low. Serologic testing is frequently used for epidemiologic or research purposes, but it is neither widely available nor standardized. The detection of specific antibody against pertussis toxin is a well-known test for its ability to detect the good immune response after *B. pertussis* infection. PT IgG ELISA test is also commonly used in the diagnosis. Compared to the other methods, PT IgG ELISA test is also commonly used in many studies for the diagnosis of *B. pertussis* infection in adults with prolonged cough regarding the sensitivity and specificity, however, there is no standardization in the interpretation until recently [28]. In the present study, when PCR and serology tests were employed for the diagnosis of pertussis infection, 18.4 % of patients with prolonged cough had evidence of acute infection of pertussis. This seems to be high, compared to the previous studies [17–20].

Undiagnosed infected adults may spread the organism to the nonimmune individuals, and the infection in some high-risk groups including young children can be more severe and potentially fatal. Thus, the awareness of pertussis in adolescents and adults with prolonged cough should be emphasized as one of the public health problems in Thailand where pertussis vaccine (combined with diphtheria toxoid and tetanus toxoid) has been part of EPI in childhood period. Antibiotic therapy such as macrolides or cotrimoxazole during the early catarrhal phase may eradicate the organism from patients’ nasopharynx, the definite diagnosis of pertussis is rarely considered at the time before initiating antibiotic treatment in prolonged cough in adolescents and adults [29]. However, Increasing clinician awareness and reporting of pertussis in patients who have prolonged cough illnesses with the use of more sensitive diagnostic techniques (especially PCR testing) may be the important management for this diseases in Thailand. The national vaccination program in Thailand must be emphasized as the role of routine pertussis vaccination in adolescents and adults as well as in infants and children to create the herd immunity in the population. Because recent studies have shown that the protection from pertussis vaccines given in childhood wanes substantially over time, tetanus-diphtheria-acellular pertussis (Tdap) vaccine should be actively provided for adolescents and adults especially with pregnancy, healthcare personnel and any persons who have close contact with infants of less than 12 months of age in many countries including Thailand [30].

This study was performed in a tertiary care center in the capital city of Thailand in limited period of time. This result might not represent the prevalence of pertussis in other regions in the country. Further research should extend to other regions in to identify the cause of prolonged cough in Thai adults and adolescent.

## Conclusion

In conclusion, the prevalence of pertussis has increased in adolescents and adults with prolonged cough in many countries. Pertussis also is being increasingly recognized as a cause of prolonged, distressing cough among adults in Thailand. The results of the present study address the need of good surveillance system and better immunization program in Thai adolescents and adults to prevent the transmission of this disease to high risk groups; such as newborn infants.

## Abbreviations

Ct: cycle threshould
ELISA: enzyme-linked immunosorbent assays
HIV/AIDS: Human immunodeficiency virus/ Acquired immune deficiency syndrome
PCR: polymerase chain reaction
PT IgG: pertussis toxin Immunoglobulin G
Tdap: tetanus-diphtheria-acellular pertussis
Tm: melting temperature
